# 

**Published:** 1891-11-21

**Authors:** 


**Notices of Births, Deaths, and Marriages**, are inserted in this column at a charge of 2s. 6d. for an announcement not exceeding four lines.

### NOTICE TO CORRESPONDENTS.

All MS., Letters, Books for Review, and other matters intended for the Editor should be addressed THE EDITOR, THE LODGE, PORCHESTER SQUARE, LONDON, W.

The Editor cannot undertake to return rejected M.S., even when accompanied by stamped directed envelope.

**Notice to Advertisers and Subscribers.**

All Advertisements, Orders for Copies of THE HOSPITAL, and Business Communications should be addressed to The Publisher, *not to the Editor*, THE HOSPITAL, 140, Strand, W.C.

The Cost of Subscription, post free, is as follows:—Per week, 1½d.; per quarter, 1s. 8d.; per half-year, 3s. 3d.; per annum, 6s. 6d. Foreign:—Per annum, 8s. 8d.; payable, in all cases, in advance.

**"Burdett's Hospital Annual," 1891—1892.**

Third year of publication. 600 pp. Boards, gilt lettering. A most complete guide to British and Colonial Hospitals, Dispensaries, Nursing and Convalescent Institutions, and Asylums. Price 3s. 6d. Published by THE HOSPITAL (Limited), 140, Strand, W.C.

**NOTICE.—The Index for Vol. X. (ending September, 1891). is on sale at the Office of this Journal, price 2½d.. post free.**

### GENERAL CHARITIES.

[This Column is devoted to an account of work of charitable institutions other than Medical Charities. The Editor will be glad to receive reports or news of work at 140, Strand. *Philanthropy* should be plainly written on the envelope.]

The Rev. Prebenday Whittington will take the chair at the annual meeting of the National Benevolent Institution, which is to be held at the Freemasons' Tavern, Great Queen Street, on Thursday, the 26th inst., at twelve o'clock.

The Marquis of Waterford appeals for donations on behalf of the **IRISH DISTRESSED LADIES' FUND.** He writes, that "the pensions and allowances have had to be largely reduced, and if it had not been for a few anonymous and most generous donations received lately there would have been every chance of this most deserving charity coming to an end. During the past half-year 140 old and delicate ladies have received pensions, 70 cases of illness have been attended to and relieved, numbers of children have been educated, and families have been assisted to emigrate. The Secretary will thankfully receive and acknowledge any subscriptions or donations at the office of the charity, 17, North Audley Street, W., which is also the depot for the sale of the Irish Distressed Ladies' work."

An evening concert, in aid of **THE INVALID CHILDREN'S AID ASSOCIATION**, is announced to take place at Queen's Gate Hall, Harrington Road, S.W., at 8.30 p.m. on Friday, the 27th inst., which we hope will be well supported. The primary aim of the Association is to find for each of its children a friend who shall do for the child, as far as possible, "everything that experience, common sense, and kindness may suggest." In addition to this, the Society strives to provide skilled nursing, medical advice, surgical appliances, &c., and admission into hospitals and homes, which are so frequently needed by children of the poor, but which they cannot obtain unaided. Those who cannot attend the concert can help a good work by sending donations to the office, 18, Buckingham Street, Strand.

During the past week a special effort has been made by the Managers of **THE ORPHANAGES OF MERCY**, 27, Kilburn Park Road, N.W., to collect the necessary funds which are urgently needed to carry on the work of the Society. Miss Ashdown will be grateful either for any sums of money or for articles of clothing to help in the work. The Society receives boys and girls from all parts of the United Kingdom who have lost both parents, and are without a friend or a penny in the world, and now has under its care over five hundred children, who, but for the existence of such institutions as this, would probably be utterly destitute. Miss Ashdown appeals further for help to maintain the Night Refuge. Here during the winter, which brings such an increase of suffering to the poor amongst whom the Church Extension Association works, homeless, sick, and helpless men, and starving children come for shelter.

The Bishop of Oxford, who presided at the annual meeting of the Oxford Diocesan Branch of the **CHURCH OF ENGLAND TEMPERANCE SOCIETY**, expressed his full sympathy with the cause. As the Bishop of Marlborough pointed out, the recent correspondence which has appeared in the columns of the *Times* shows that there is a healthy opinion in the country with regard to the temperance movement. Most people, who have any brains with which to think, are beginning to recognise the fact that it is the custom to take far more stimulants than are necessary for the maintenance of a healthy and vigorous frame. The difficulties in the way of removing the evils which injure our national life, and which by bringing misery into so many homes cause the degradation of numerous individual lives, have been and are likely to continue to be great; but they are not insurmountable, as the progress made during recent years in the education of the public on the subject tends to prove.

The Committee of the **ASYLUM FOR IDIOTS**, Earlswood, Redhill, are naturally anxious to secure the legacy of eight hundred pounds bequeathed to the Asylum conditionally by the late Rev. Peter Southmead Glubb. The Institution cannot participate in the provisions of the will unless it is able to produce indisputable proof that the legacy has been the means of obtaining fresh donations to meet the gift and doubling the sum. No doubt Mr. Glubb's object was to make his conditional bequest as advantageous to the Institution as possible, and the Treasurer and Secretary therefore appeal urgently to the public for donations towards the sum needed to enable them to claim the legacy. They urge their claim for support on the ground that "the Institution is very straitened for means to maintain the large number of 627 patients at present under its care and instruction." As we have previously pointed out, the idea of an idiot does not commend itself to many people in a pleasing aspect, and they are apt to think that idiots have no faculty for useful work, and so fail to justify their existence. A visit to Earlswood would speedily disabuse their minds of this false view of the capacity of the idiot. Many of the patients follow a handicraft, by means of which they are enabled to earn a living and to return to their homes without being, as formerly, useless burdens. It is a fact, that in the mat-making industry they are in a position to compete in the open markets. Thus, by the work of its inmates, Earlswood is made a partially self-supporting institution. Donations sent to the Secretary, 36, King William Street, E.C., will be gratefully acknowledged.

### SCRAPS AND GLEANINGS.

There are 2,000 cases of influenza at Monte Video.

Croydon Hospital Saturday collection stands at upwards of £453.

During the year nearly 20,000 in and out-patients have been relieved at Sir Patrick Dun's Hospital, Dublin.

General Feilden has presented to the Blackburn Infirmary a sum of £175 for the New Nurses' Home Fund.

The quarterly meeting of the Newcastle Royal Infirmary revealed a serious deficiency in the finances of the hospital.

Several malignant cases of diphtheria are reported at Dunmow. Of three cases in one cottage, two have proved fatal.

It is proposed to give a testimonial to Mr. Smedley, Secretary, for his valuable services to the Birmingham Hospital Saturday Fund.

A Glasgow citizen, fired by the result of the Liverpool Hospital Saturday Collection, proposes a similar fund should be started in the first-named city.

The Duchess of Connaught distributed the prizes at the Portsmouth High School for Girls last week. The Duke was in the chair.

A donation of £500 has been offered by one gentleman towards an eye infirmary at Greenock. The present room in the Municipal building is far too small.

We are glad to see that the Spinal and Orthopædic Hospital, Birmingham, has been very successful this year, and has collected thirty-eight new subscribers.

The Baroness Burdett-Coutts presided on the 13th inst. at a drawing-room meeting, held at the house of Mr. and Mrs. Whitman, Hampstead in aid of the North London Hospital.

Princess Christian has kindly undertaken to open a bazaar at the Queen's Gate Hall, on December 2nd, to raise funds for free breakfasts for children, and dinners for the poor during the coming winter.

A bazaar was opened last week at Preston, in aid of the Fund for the erection of a Poor Law certified Roman Catholic School for Lancashire. Land has been purchased outside Preston at a cost of £4,510.

St. Paul's Eye and Ear Hospital, Liverpool, received 474 cases during the year as in-patients, and a large number of out-patients. It is significant that patients' payments amounted to no less a sum than £500.

The Lord Mayor and the Lady Mayoress have become members of the General Committee of the Irish Distressed Ladies' Fund, and his Lordship will be glad to receive any donations for the charity at the Mansion House.

Mr. John Magee, aged sixteen, has found his way into the Cardiff Infirmary with fifty-three marbles inside him. He appears to have swallowed them solely to win a wager. He is said to be none the worse for his stony meal.

Dr. B. W. Richardson opened a working man's club near Oxford Street, and pointed out that such institutions would become more necessary and popular as hours of labour were shortened, as they appeared likely to be.

In the annual report of the Brackley Cottage Hospital it is noted that the custom which obtains at the institution of feeding convalescent patients as well as possible is proved by experience to be the most economical in the end.

It is proposed by the Bishop of Llandaff to dedicate the Missions to Seamen Church at Cardiff on Wednesday next, the 25th inst. The church has cost, with the site and stone, which were given free, about £11,000. About £2,000 remains to be raised.

The Derbyshire Hospital for Sick Children presents a favourable report. Expenses are slightly greater than previous years, but that the expenditure has not been an unnecessary one we feel confident as the hospital is most carefully managed.

We are sorry to learn that the finances of Sir Patrick Dun's Hospital, Dublin, are in a bad way. The hospital used to receive an average income of £1,230 from Sir Patrick Dun's estates. Last year they received only £200, nor are matters likely to improve at present.

Some alarm is arising at Huntley. It appears that the County Council propose taking over the Cottage Hospitals for motives of economy, it is said. To do this, the institution must be converted into an infectious diseases hospital, as the rates can only be applied to such an institution.

At the annual meeting of the Bridgwater Infirmary it was justly pointed out that it is a serious loss to a hospital if bequests, however generous, tend to lessen the amount of subscriptions received. The whole vitality of a hospital depends on a steady annual subscription list.

A panic was caused in Prague last week by a fire which broke out at the convent in that city. The convent has a large hospital for incurables, and these patients were carried into the garden in their beds. The Abbess had reprimanded a gardener who set fire to the premises for revenge.

The *Globe* tells us that Paris now rejoices in a lady chemist. She is quite properly qualified, and if she takes care that her assistants are equally competent, all may be well. Why women should not adopt this profession we know not. Their painstaking and conscientious qualifications are just those most needed for such work.

Princess Christian visited two praiseworthy operations of the London School Board—those connected with economical cookery and efficient needlework. The London Vegetarian Society and the Council of the Bread and Food Reform gave a sample halfpenny dinner for 300 children. The needlework shown was pronounced excellent.

It is stated that nearly the whole of the passengers and crew of the P. and O. steamer *Massilia*, which arrived in the Albert Dock from Australia, having suffered severely from influenza. At one time during the voyage ninety-six persons were prostrate. The unfortunate passengers of the *Massilia* are supposed to have contracted the epidemic at an entertainment at Melbourne shortly before the ship started on her voyage. Careful watching of influenza certainly points to the fact that large assemblages tend to spread the disease more rapidly than any other condition.

### THE STUDENT OF MEDICINE.

[All communications for this Supplement should be addressed to THE EDITOR of THE HOSPITAL, at 140, Strand, with the words, "Students' Column," written in the left hand top corner of the envelope.]

### APPOINTMENTS.

At the North-West London Hospital, Kentish Town Road, J. H. Fowler, M.R.C.S., L.R.C.P., has been appointed Senior, and C. D. Sutherland, M.B., M. C. Edin, M.R.C.S., junior, resident medical officer. At the City of London Hospital for Diseases of the Chest, Victoria Park, W. J. Hadley, M.D., M.R.C.P., F.R.C.S., has been appointed assistant physician. At the Middlesex Hospital, Archibald Kidd, M.R.C.S., L.R.C.P., has been appointed house physician. At Guy's Hospital, D. S. Long, M.B., B.C.Camb., has been appointed assistant house physician. At the Royal Alexander Hospital for Sick Children, E. L. Lortain, B.A., M.B., has been appointed house surgeon. At the Walsall Cottage Hospital, W. Poole, M.R.C.S., has been appointed house surgeon. At the Queen's Hospital, Birmingham, Ernest A. Sadler, M.B.Lond., M.R.C.S., has been appointed house physician. At the Hospital for Diseases of the Throat, Golden Square, Henry Sharman, M.B.Lond., M.R.C.S., L.R.C.P., has been appointed resident medical officer. At King's College Hospital, Mansfield Knox Soutter, M.R.C.S., L.R.C.P., has been appointed house surgeon. At the Paddington Green Children's Hospital, H. G. Adamson, M.B.Lond., M.R.C.S., L.R.C.P., has been appointed house surgeon. At the Royal Isle of Wight Infirmary and County Hospital, Ryde, R. A. Bayliss, M.R.C.S., L.R.C.P. has been appointed house surgeon and secretary. At the Warrington Infirmary, Ernest E. Bowden, M.R.C.S., L.S.A., has been appointed senior house surgeon. At the Cheltenham General Hospital, H. Boyd Cardew, M.R.C.S., L.R.C.P., has been appointed house surgeon. At the Royal Maternity Hospital, Edinburgh, C. C. Douglas, M.B., C.M.Edin., has been appointed house surgeon. At the Bethlem Hospital, A. Ellis Durham, M.A., M.B., B.C. Camb., has been appointed resident clinical assistant. At the Belfast Union Hospital, R. A. Gardner, M.B., C.M. Glas., has been appointed house surgeon. At the Manchester Southern and Maternity Hospital, William Griffith, M.B., Ch.B. Vic., has been appointed house surgeon. At the Great Northern Central Hospital, A. Anthony Howell, M.R.C.S., L.R.C.P., has been appointed casualty officer. At the Richmond Hospital Jacob Arthur Nowell L.R.C.P., L.R.C.S., has been appointed Hon. Medical Officer to see out-patients.

### VACANCIES.

The following vacancies are announced this week, the amount of salary in each case being given after the name of the institution:

### Resident Medical Officers.

City of London Hospital for Diseases of the Chest, Victoria Park, House Physician—Board and residence, &c.
Darlington Hospital and Dispensary, House Surgeon—Salary £100, with board, &c.
North-Eastern Hospital for Children, Hackney Road, Junior House Surgeon—Salary £60, with board, &c.
Northern Infirmary, Inverness, House Surgeon and Apothecary—Salary £100, with board, &c.
Paddington Infirmary, Resident Clinical Assistant—Honorarium of 12 guineas for six months, with board, &c.
Royal Hospital for Diseases of the Chest, City Road, House Physician—Salary £40, with board, &c.
Taunton and Somerset Hospital, Taunton, Assistant House Surgeon.
West London Hospital, Hammersmith Road, House Physician, also House Surgeon—Board, &c.

### Non-Resident Medical Officers.

City of London Hospital for Diseases of the Chest, Pathologist—Salary £100.
London Hospital, Medical Registrar—Salary £100.
Owens College, Manchester, Professor of Pathology—Salary £650.
Royal Hospital for Diseases of the Chest, City Road, Assistant Physician.

### NOTES FROM THE SCHOOLS.

### Edinburgh University.

The session opened on Tuesday, Oct. 13th, with the customary inaugural lectures delivered by the professors.

The MacEwan Hall for graduation ceremonies, &c., is approaching completion; the finishing of this hall will complete the design of the university new buildings.

The managers of the Royal Infirmary are considering the advisability of adding 200 beds to that building for one of the new wards to be set apart for the lady medicos.

The Scottish Students' Long-book has run through the first edition, which was only out at the end of last session, and a new edition in various forms is in preparation.

It is not many students' medical societies that can boast of

### The Hospital Advertiser.

MEDICAL APPOINTMENT.

THE HOSPITAL FOR SICK CHILDREN,

### THE STUDENT OF MEDICINE—(*continued*).

such a long record as the Royal Medical Society. Their 155th session was formally opened on October 23rd, by William McEwin, Esq., LL.D., &c., of Glasgow, with an inaugural address.

Mr. A. J. Balfour, M.P., has been elected Chancellor of the University in the place of the late Lord Glencorn.

### University of Durham Musical Society.

The first of a series of five smoking concerts was held in the Assembly Rooms of the County Hotel, Newcastle, on Saturday, October 31st, with Dr. Howden in the chair. There was a large and enthusiastic audience, and a good programme had been provided by the energetic secretaries, Messrs. Peacock and Hodgson. Mr. Daniel gave a spirited rendering of "The King's Own," the audience taking up the chorus. Molley's "Lighthouse Keeper," was given by Mr. Hey, who was followed by Mr. W. E. Jones with "Good Company," in which his tenor voice showed to advantage. "Wot Cheer," by Mr. Worth, was enthusiastically received and encored. In response to a demand for an encore for Mr. Simpson's "Sailor's Story," he and Mr. Stokoe gave the duet "Excelsior." This was the best item in the programme, and was rendered most artistically and effectively, both performers having good voices. Mr. Thorpe concluded the first part by singing "That's How He Carries On," with "It's a Pity to Waste It" as an encore. Mr. Elam sang a drinking song, and Mr. Visser then gave "It's Another Colour Now," and "Yah, Yah!" as an encore. "The Powder Monkey," with its chorus of "Soon We'll Be In London Town," was sung by Mr. Coleman. In the absence of Mr. Gowley, Mr. Clark gave a humorous recitation on the subject of "Love-making," followed by a characteristically Scotch anecdote. Messrs. Daniel and Jones then sang Braham's duet, "Love and War," with good effect. A song by Mr. Richardson was followed by "The Little Vagabond Boy," by Mr. Thorpe, who was again very well received. A pleasant evening was brought to a close with the singing of the National Anthem.

### Westminster Hospital Students' Dinner.

This annual event took place on Saturday, at the Criterion, Piccadilly. Dr. C. E. A. Macleod occupied the chair. A most pleasant evening was passed. At the conclusion of the dinner the usual toasts were got through—"The Queen and Royal Family," proposed by the Chairman; Dr. Cullinan proposed "The Health and Success of the Hospital Medical School and Lectures"; "The Present Students," by Dr. E. C. Macleod; "The Past Students," by Mr. Nitch-Smith," "The Ladies," by Mr. Cotton; "The Visitors," by Mr. Churchill. Mr. Dodd responded. "Sports," by Dr. Bell. "The Health of the Chairman" was proposed by Mr. Wilfred Fleming, and drunk with musical honours. Several good songs were ably rendered. The whole affair was a great success, praise being due to Mr. Nitch-Smith, the Honorary Secretary to the dinner.

### King's College, London—October, 1891.

The entrance scholarships and exhibitions have been awarded as follows: Warneford Scholarship (Class I.): Cowie, £75; Ross, £50. Sambrooke exhibitions; Clifford, £60; Lansdown and Schaub, £20 each. Science exhibitions: Jacobs, £60; R. T. Williams, £40.

### Sheffield General Infirmary.

Mr. H. B. Lee, who has been tutor to the Sheffield Medical School during the last ten years, has resigned in favour of private practice. Mr. Francis Stevens, M.R.C.S. England, L.R.C.P. London, has been appointed in his place.

---

### PASS LISTS.

### Examining Board in England by the Royal Colleges of Physicians and Surgeons.

The following gentlemen passed the Second Examination of the Board in the subjects indicated at a meeting of the Examiners on the 7th inst.:—

Anatomy and Physiology.—C. P. Allen, student of Middlesex Hospital and Mr. Cooke's School of Anatomy and Physiology; J. C. Constable, of Guy's Hospital; G. Cole, of King's College; W. Craven, of St. Mary's Hospital; J. S. Mackintosh, of St. Bartholomew's Hospital; E. W. Robinson, of Westminster Hospital.

Anatomy only.—J. Heard, of Westminster Hospital; P. H. Court, of Middlesex Hospital; H. Harvey and H. Harrison, of London Hospital; M. Carter, of Charing Cross Hospital.

Physiology only.—A. P. Dantes, of Grant Medical College, Bombay; F. A. L. Hammond, of Charing Cross Hospital; H. S. Revell, W. B. Orme, and T. Rhind, of University College; J. C. Young, of Buffalo College and Mr Cooke's School of Anatomy and Physiology; E. S. Roberts, F. A. Evison, and H. E. Sharman, of Guy's Hospital; E. W. Gregor and A. H. P. Crosby, of Middlesex Hospital; F. R. Gibbs and R. D. Evans, of St. Mary's Hospital.

Passed on the 8th inst.:—

Physiology only.—W. C. Hutley, M. N. J. Rigby, W. Roydon, and T. Barker, of St. Bartholomew's Hospital; W. H. Horton, of St. Bartholomew's Hospital and Mr. Cooke's School of Anatomy and Phy-

### THE STUDENT OF MEDICINE—(*continued*).

siology; W. S. Webb, F. J. Loveday, and C. J. P. T. Gibbons, of London Hospital; M. J. Longinotto and W. Pomeroy, of St. Thomas's Hospital; S. S. Wallis, T. M. Nicholson, and W. W. Henson, of Guy's Hospital; P. Best, of University College; F. V. Denne, of Westminster Hospital and Mr. Cooke's School of Anatomy and Physiology; A. Stockton, of King's College.

The following gentlemen, having passed the necessary examinations, and having conformed to the bye-laws and regulations, were at the quarterly meeting of the Council on Thursday, the 15th inst., admitted Members of the College:—K. F. T. Buée, L.S.A., The Cedars, Slough; D. E. Jones, M.B. Melb., Melbourne, Victoria; C. F. Pridham, L.R.C.P. Lond., "Digbys," Exeter; A. C. Rendle, L R.C.P. Lond., Devonport.

The following gentlemen, having previously passed the necessary examination, and having now attained the legal age (twenty-five years), were at the same meeting admitted Fellows of the College: H. Calger, L.R.C.P.Lond., Brownswood Park, Green Lanes, N. Diploma of Member dated February 13th, 1890. H. J. Waring, M.B.,Lond., L.R.C.P. Lond., St. Bartholomew's Hospital. Diploma of Member dated May 8th, 1890.

### Society of Apothecaries of London.

The following candidates passed in the respective subjects during the present month:—

Surgery.—G. H. Baird, Dublin; C. E. Dawes, F. B. Lewis, and A. R. Walters, London Hospital; W. P. Hilliam, Sheffield; A. E. Leitch, Liverpool; A. W. Read and H. G. White, St. George's Hospital; E. A. R. Covey, St. Bartholomew's Hospital; S. A. E. Griffiths, Middlesex Hospital; W. C. Howle, Queen's College, Birmingham; W. A. Williams, Calcutta and Middlesex Hospital.

Medicine, Forensic Medicine, and Midwifery.—W. H. Cooke, St. George's Hospital; A. B. Hargraves, London Hospital; S. Nesfield, Manchester; A. T. Duka, Cambridge University and St. George's Hospital; A. B. Harman, King's College; H. H. L. Patch, St. Thomas's Hospital.

Medicine and Forensic Medicine.—W. C. Howle, Queen's College, Birmingham.

Medicine and Midwifery.—A. L. Wykham, Washington.

Medicine. R. W. Brimacombe, St. Mary's Hospital; C. H. A. Maggs, Charing Cross Hospital; C. M. O'Brien, Newcastle-on-Tyne.

Forensic Medicine and Midwifery.—W Ashby, Guy's Hospital.

Forensic Medicine.—T. H. English, London Hospital; C. P. Morgan and C. F. Warren, Guy's Hospital.

Midwifery.—S. R. Lane, Middlesex Hospital.

To Messrs. Covey, English, Harman, Leitch, Morgan, Reid, Warren, and Williams was granted the Diploma of the Society, entitling them to practice Medicine, Surgery, and Midwifery.

### University of Oxford.

At a Congregation held on the 10th inst. the Degree of M.D. was conferred on George B. Longstaff, New.

### University of Cambridge.

The following is a list of gentlemen examined and approved in both parts of the examination in State Medicine: F. W. Andrewes, E. Antrobus, L. A. Baine, J. M. Beamish, C. Beesley, R. Bird, J. F. Braga, J. Carroll, F. W. Clark, H. Cooper-Pattin, R. W. Coppinger, B. H. Deare, M. A. Dutch, W. W. E. Fletcher, R. N. Goodman, F. Grange, W. H. Hamer, T. H. Harwood, Richard Jones, W. H. Kelson, D. Lawrie, H. J. Lotz, J. C. M'Clew, R. Miller, G. E. Moffet, H. B. Osburn, J. Penny, C. Porter, J. Rannie, C. G. Robson-Scott, T. D. Savill, Edmund M. Smith, J. E. Squire, G. N. Stathers, G. T. Tate, Arthur Thomson, J. A. Turner, G. Wale, G. E. Walker, H. H. White, E. Wilkinson, W. G. Willoughby, A. S. Wilson, G. Wilson, and C. W. F. Young.

At a Congregation held on October 22nd the following Degrees were conferred:—

Doctors of Medicine.—Archibald Keightley, Pembroke; Edward Petronell Manby, Cavendish Hostel; Ernest Lloyd Jones, Downing.

Batchelors of Medicine and Batchelors of Surgery.—Arthur Hugh Thompson, Trinity; Frank Mortimer Rowland, Gonville and Caius.

At a Congregation held on the 14th inst., the following Degrees were conferred:—

Doctor of Medicine.—William Arthur Evelyn, Gonville and Caius.

Batchelors of Medicine and Batchelors of Surgery.—Arthur Geo. Phear, Trinity; Robert Curzon Mollison Colvin-Smith, Gonville and Caius.

### University of Edinburgh.

The following gentlemen received the degrees of M.B., C.M., on October 14th: F. T. Auden, Shrewsbury; F. R, K. Ball, Edinburgh; T. W. Banks, Edinburgh; E. A. Braithwaite, Cumberland; S. B. J. Bulteel, Devon; P. G. Van der Byl, Cape Colony; D. Campbell, Lancashire; G. C. Cathcart, Edinburgh; J. A. H. Duncan, Inchture; M. C. Evison, Lancaster; R. S. Ferguson, Beamish; S. B. Figgis, Brighton F. D. Fisher, Workington; G. Fleming, Edinburgh: J. W. Forbes, Dundee; F. W. Foxcroft, Cheshire; D. C. Gardiner, Essex; H. C. Garth, Kennington; W. E. Godber, Edinburgh; C. J. Gomes, Demerara; G. T. Guild, Newburgh; E. B. Gwynn, Shifnal; H. H. Hannay Leamington; W. B. Harry, Llanelly; H. B. Knox, Kingston, Jamaica M. K. Khan, Hyderabad; W. L. Legg, Scarborough; A. A. McLeod, Greenock; Duncan McNeill, Edinburgh: A. K. Melville, Edinburgh; C. A Morgan, Holywell, Flints; H. B. T. Morgan, Carnarvon; D. S. Morrison Argyllshire; E. R. Parry, Edinburgh; S. P. Peart, Edinburgh; J. L Reed, Sydney, Australia; Stanley Riseley, Bristol; A. F. Rosa, Edinburgh; John Sharp, Boat of Garten; W. J. J. Stewart, Lanark; F. A. Symons, Halifax, N.S.; F. M. Thomas, Liverpool; F. M. Thomson, Lasowade, N.B.; A. G. Thompson, Portobello, N.B.; J. T. Thyne, Edinburgh; Hubert Tibbits, Warwick; F. B. Turton, Edinburgh; J. C. Whyte, Edinburgh; E. S. Yonge, N. Devon.

### THE STUDENT OF MEDICINE—(*continued*).

### University of Glasgow.

The following candidates have passed the Third Professional Examination for the Degrees of Bachelor of Medicine and Master of Surgery :—

Including Pathology.—W. H. M. Alexander, J. E. Bow, W. B. Brodie, W. M. Brown, A. P. Campbell, D. B. Campbell, R. J. Carroll, J. Carslaw, M.A., J. S. Davies, M.A., W. W. Don, A. R. Ferguson, R. Guy, J. B. Hartley, A. Henderson, C. Highet, H. E. Jones, T. Kirkwood, M.A., T. D. Laird, H. Lawrie, H. C. Marr, J. Moffat, J. D. R. Monro, M.A., J. Morton, J. H. MacArthur, A. H. M'Cracken, J. M'Donald, M.A., G. M'Feat, M. Macnicol, M.A., A. S. M'Pherson, W. M'Walter, R. E. Newton, J. Paterson, M.A., W. J. Richard, M.A., J. J. Robb, A. Robertson, M.A., J. A. Robertson, R. Taylor, J. H. Teacher, M.A., A. Wauchope, A. White, C. Wilson, W. S. Young.

Not including Pathology.—P. Aitken, J. Anderson, C. J. Babes, J. J. Boyd, F. Brown, G. B. Buchanan, B.A. Cantab., J. W. M. Buick, W. Campbell, J. J. Carruthers, D. Charters, R. R. Coyle, J. Crawford, J. Cross, D. A. Dewar, A. S. Dick, W. Duncan, W. Fulton, H. N. Gardiner, F. Gracie, P. N. Grant, T. Hamilton, J. L. Howie, N. S. Jeffrey, G. L. Kerr, W. A. Kirkwood, R. A. E. Lowry, D. R. Miller, E. J. Morris, W. M'Nivan Muat, J. M'Kay, G. M'Laughlan, P. C. MacRobert, E. L. Pollonais, R. Ramsay, L. A. Rowden, T. L. Shields, A. R. Smith, J. Smyth, C. Stewart, G. N. Turner, J. Whitehouse, T. Wright, A. A. Young, M.A., J. C. Young.

### University of Durham : Faculty of Medicine.

At the Second Examination for the Degree of Bachelor in Medicine the following candidates satisfied the Examiners :—

Second Class Honours.—R. A. Morris, College of Medicine, Newcastle-on-Tyne.

Pass List.—P. R. Ash, Yorkshire College, Leeds ; R. A. Bolam, T. M. Clayton, E. D. Dingle, W. E. Gibbs, K. C. Hill, J. M. Hope, and R. W. Morgan, all of the College of Medicine, Newcastle-on-Tyne ; P. Coleman, M.R.C.S. Eng., L.R.C.P. Lond., St. Thomas's Hospital ; W. F. Fisher, London Hospital ; J. J. Grace, Otago, New Zealand ; N. C. Gwynn and R. C. Leonard, both of the Bristol Medical School ; G. Jimenez, Guy's Hospital ; A. C. Leigh, University College, London ; L. G. C. Vintras, M.R.C.S., L.R.C.P. Lond., B.Sc. Paris, St. Mary's Hospital.

The following candidates were successful at the examinations indicated :—

Degree of Bachelor in Hygiene.—T. Buckham, M.B., B.S., and W. H. Turnbull, M.B., B.S., both of Durham.

Licence in Sanitary Science.—F. W. Gunn, M.R.C.S., L.R.C.P., L.S.A.

### ATHLETICS.

### Football.—Rugby, October 24th.

*Westminster Hospital* defeated the *Dental Hospital*, at Raynes Park, by two tries to nil.

### October 30th.

*Owens College, Manchester*, were beaten by *Blackley* by two goals and three tries to nil. *St. Mary's Hospital* were beaten by *Charlton*, at Charlton, by two goals and three tries to a try. *University College Hospital* were beaten by *Hampstead*, at Park, by a try to nil. *Guy's Hospital* drew with *Kensington* at Stamford Bridge. The game throughout was very fast and rather rough, but neither side succeeded in scoring a point. Three of the Hospital men were injured, one of them having his nose broken. *Middlesex Hospital* were beaten by *Royal Military College* at Sandhurst. The match was played before a large gathering of people in fine weather. The Hospital played up well and determined, but nevertheless they were unable to score more than a try, and were defeated by three goals and two tries to a try.

### Association.

*London Hospital* drew with *Tottenham*, each side scoring a goal. *Guy's Hospital* beat *Luton*, at Luton, by four goals to one. *Royal Veterinary College* beat *North-West Rangers*, at Regent's Park, by three goals to nil. *St. Thomas's Hospital* were beaten by *West Kent*, at Chislehurst, by four goals to nil, after a very good game.

### Rugby, November 7th.

*St. Bartholomew's Hospital* were beaten by *Wickham Park*, at Lee, by four goals and three tries to nil. The hospital played two men short. *St. George's Hospital* were beaten by *Wasps*. at Castle Hill, by a goal and three tries to nil. *Royal Veterinary College* were beaten by *Eltham House* by a goal and three tries to nil. *London Hospital* second were beaten by *Park House*, at Blackheath, by four goals and two tries to nil. *The London Hospital* were beaten by *Portsmouth* by a goal and a try to nil. *St. Thomas's Hospital* were beaten by the *Royal Military College* at Sandhurst by goals and one try to a try. The match was played in beautiful weather. The home team kicked off and some pretty passing was witnessed on both sides. Sandhurst then obtained a try which was converted by Lloyd into a goal. Directly after half-time Thomson kicked a good goal, and soon after he dropped a second one. *Guy's Hospital* were beaten by *Swansea* at Swansea by 23 points to nil. In the first half Whapham and Coke obtained tries, and Bancroft dropped a goal, afterwards Deacon, London, and James got over, and Bancroft nearly kicked another goal from half-way.

### Association, November 7th.

*St. Thomas's Hospital* were beaten by *Brentwood* at Brentwood, by five goals to two. *Guy's Hospital* were beaten by *Eastbourne* by six goals to two. *St. Bartholomew's Hospital* beat *Vampires* at Dulwich by seven goals to one. *St. Mary's Hospital* beat *Condors* at Wimbledon by six goals to nil. *Royal Veterinary College* beat *Millwall Athletic Reserves* on the latter's ground by two goals to nil.

